# Association between laboratory capacities and world-cup performance in Nordic combined

**DOI:** 10.1371/journal.pone.0180388

**Published:** 2017-06-29

**Authors:** Vegard Rasdal, Ronny Fudel, Jan Kocbach, Frode Moen, Gertjan Ettema, Øyvind Sandbakk

**Affiliations:** 1 Centre for Elite Sports Research, Department of Neuromedicine and Movement Science, Factulty of Medicine and Health Science, Norwegian University of Science and Technology, Trondheim, Norway; 2 Institute of Movement and Training Science in Sports, Faculty of Sport Science, Leipzig University, Leipzig, Germany; 3 Department of Education, Norwegian University of Science and Technology, Trondheim, Norway; University of Debrecen, HUNGARY

## Abstract

**Background:**

Nordic combined (NC) is an Olympic winter-sport performed as a ski jumping (SJ) event followed by a cross-country (XC) pursuit race employing the skating style.

**Purpose:**

To elucidate the associations between sport-specific laboratory capacities and SJ, XC skiing, and overall NC performance in a world-cup NC event.

**Methods:**

Twelve international world-cup NC athletes from 8 nations performed laboratory testing one day prior to participating in a world-cup NC event. Squat jumps and SJ imitations (IMIT) were performed on a three-dimensional force plate, whereas XC skiing-specific physiological characteristics were obtained from roller ski skating tests on a treadmill and an all-out double poling (DP) test. Finally, body composition was measured. Laboratory capacities were correlated against performance in SJ, 10-km XC skiing, and overall NC in the world-cup event. Multiple regression analysis was used to determine the best suited laboratory variables for predicting performance.

**Results:**

Vertical IMIT velocity together with body-mass provided the best prediction for SJ performance (r^2^ = 0.70, p<0.01), while body-mass-normalized $\dot{V}O_{2\mathrm{peak}}$ and DP power provided the best prediction for XC performance (r^2^ = 0.68, p<0.05). Body-mass-normalized $\dot{V}O_{2\mathrm{peak}}$ was the only significant correlate with overall NC performance (r^2^ = 0.43, p<0.05) in this competition.

**Conclusion:**

Overall, the concurrent development of $\dot{V}O_{2\mathrm{peak}}$, upper-body power, and SJ-specific vertical jump capacity while minimizing body-mass within the BMI limit set by FIS should be considered in the seasonal training of NC athletes.

## Introduction

Nordic combined (NC) is a traditional Olympic winter-sport, and is performed as a ski jumping (SJ) event followed by a cross-country (XC) pursuit race employing the skating style over a distance of 5–15 km (standard competition is 10 km). Both events are carried out on the same day with 1–3 hours in between, where each athlete starts the XC race with a time disadvantage per point lost to the winner of the SJ event. Consequently, NC athletes need to perform well in two fundamentally different sports; SJ that requires well-developed explosiveness and jumping technique and XC skiing where aerobic energy delivery and skiing efficiency are key determinants [1–4].

Of the different phases of a ski jump (i.e. in-run, take-off, flight phase, landing), the take-off is regarded as the most crucial for performance due to its influence on the initial vertical velocity of the flight and the maintenance of high horizontal velocity in the early flight phase [1, 2, 5]. In successful ski jumpers, a high vertical jump ability and a low body-mass are well-established characteristics [1, 2, 6, 7]. These characteristics also differentiates NC athletes from specialist ski jumpers [8, 9]. However, no research to date have investigated associations between sport-specific laboratory capacities and field performance in SJ among NC athletes that concurrently develop their aerobic capacity and upper-body power.

XC skiing races are performed in varied terrain and more than 50% of the racing time is normally spent in uphill terrain, which also constitutes the most performance-differentiating terrain [3, 10–12]. Accordingly, XC skiers have possessed some of the highest maximal oxygen uptake ($\dot{V}O_{2\max}$) values ever reported [3, 13–16]. Following the higher maximal aerobic capacity, better skiers also endure lower physiological stress, ski more efficiently, and produce longer cycle lengths at submaximal speeds than lower-level skiers [4, 17, 18]. In addition, more focus in recent literature has been placed on the importance of upper-body power for XC performance [17, 19, 20]. The significance of these factors for performance in NC events, however, has not been investigated. Since NC athletes may compensate lower XC skiing level with better SJ performance, they present a more heterogeneous group of endurance athletes than XC skiers [8].

The aim of this study was to elucidate the associations between sport-specific laboratory capacities and performance in SJ, XC skiing, and overall NC in a world-cup event among international NC athletes. Our major hypotheses were that $\dot{V}O_{2\mathrm{peak}}$ and vertical velocity achieved during ski jump imitations were the main correlates of overall NC performance, with upper-body power and body-mass being additional correlates of XC skiing and SJ performance, respectively. A secondary purpose of the study was also to provide benchmark values of laboratory capacities of world-class athletes in NC.

## Materials and methods

The study was approved by The Norwegian Data protection Authority. All participants signed an informed consent from before the experiment and were made aware that they could withdraw from the study at any point without providing an explanation. The study was conducted in accordance with the Declaration of Helsinki.

### Participants

Twelve international world-cup NC athletes from 8 nations volunteered to participate in the study. The participants´ age, anthropometrics, body composition, and performance level in SJ, XC skiing, and overall world-cup standing at the time of the study, classified according to the system proposed by the International Ski Federation (FIS) (www.fis-ski.com), are depicted in Table 1.

**Table 1:** Anthropometrics, body composition, and FIS ranking/world-cup standing of the twelve international Nordic combined world-cup athletes and benchmark values for subgroups of the top 3 FIS ranked athletes in cross-country skiing (XC_top3_) and ski jumping (SJ_top3_). All variables are presented as mean ± SD (range) for each group.

Variable	All (n = 12)	XC_top3_ (n = 3)	SJ_top3_ (n = 3)
Age (yr)	24.1 ± 3.7 (18–30)	27.3 ± 3.1 (24–30)	23.7 ± 2.1 (22–26)
Body height (cm)	178.4 ± 6.0 (170–187)	180.5 ± 5.41 (174.5–185)	172.8 ± 3.82 (169.5–177)
Body mass (kg)	65.8 ± 6.3 (56.5–73.1)	69.2 ± 4.42 (64.1–72.2)	59.4 ± 3.67 (56.5–63.5)
Body mass index (kg·m^-2^)	20.6 ± 0.8 (19.3–22.1)	21.2 ± 0.24 (21.1–21.5)	19.9 ± 0.36 (19.6–20.3)
Fat mass (kg)	4.2 ± 1.2 (2.3–6.7)	4.9 ± 1.57 (3.8–6.7)	3.3 ± 1.0 (2.3–4.3)
Fat mass (%)	6.3 ± 1.5 (4.0–9.3)	7.0 ± 1.9 (5.9–9.3)	5.6 ± 1.8 (4.0–7.6)
LM upper-body (kg)	33.8 ± 3.3 (27.9–38.3)	35.0 ± 2.24 (32.6–37.0)	30.5 ± 2.70 (27.9–33.3)
LM upper-body (%)	51.4 ± 1.2 (48.9–52.8)	50.6 ± 1.5 (48.9–52.0)	51.4 ± 1.7 (49.4–52.4)
LM lower-body (kg)	19.4 ± 2.1 (16.4–21.9)	20.4 ± 1.77 (18.4–21.8)	17.7 ± 1.33 (16.4–19.0)
LM lower-body (%)	29.5 ± 0.7 (28.2–30.4)	29.4 ± 0.8 (28.7–30.2)	29.8 ± 0.7 (29.0–30.4)
FIS rank ski jumping^1^	6.5 ± 1.75 (4–9)	5.0 ± 1.0 (4–6)	8.7 ± 0.58 (8–9)
FIS rank cross-country^1^	6.9 ± 2.26 (3–10)	9.3 ± 0.58 (9–10)	6.0 ± 2.0 (4–8)
FIS WC standing^2^	29.5 ± 20.3 (2–66)	15.7 ± 9.3 (8–26)	17.3 ± 15.0 (2–32)

LM = lean mass; FIS = International ski federation

^1^ FIS ranking between 1–10 based on respectively ski jumping and cross-country skiing performance in Nordic combined world cup events where 10 is highest performance level.

^2^ FIS World cup leaderboard standing in the 2013/2014 season prior to the study. Lower number is better.

### Overall design

The athletes performed a set of laboratory tests one day prior to participating in a world-cup event. SJ imitations (IMIT), and squat jumps (SQJ) to measure true vertical jump capacity, were performed on a three-dimensional force plate, whereas XC skiing-specific characteristics were obtained from submaximal and maximal roller ski tests in G2 skating on a treadmill as described in detail in a previous study [21]. In addition, body composition was determined and a 30-sec all-out double poling (DP) test was performed on a DP ergometer as a measure for upper-body power capacity. Laboratory capacities and selected anthropometrics were correlated against performance in SJ, XC, and overall NC in the subsequent world-cup competition. In addition, benchmark values of laboratory capacities and selected anthropometrics are presented for the top 3 ranked SJ (SJ_top3_) and XC skiers (XC_top3_) in the group, based on their FIS ranking. These two performance groups did not overlap.

### Methodology

To measure the magnitude and direction of forces during SQJ and IMIT jumps, two Kistler force platforms (Kistler 9286AA, Kistler Instrument Corp, Winterthur, Switzerland) were set up in series, so the athletes could place the forefoot on one platform and the rear foot on the other while performing a jump. As the ski-athlete system with bindings and SJ boots limits the plantar flexion at take-off in the hill, this setup was performed to allow for performance measures when the whole foot is in contact with the force plate during IMIT push-offs.

All treadmill tests were performed on a 5x3 m motor-driven treadmill (Forcelink B.V., Culemborg, The Netherlands) and the skiers used their own poles (90±1% of body height) using special carbide tips. All subjects were secured to the roof with a safety harness during testing. To minimize roller resistance variation, all subjects used the same pair of skating roller skis with standard wheels (IDT Sports, Lena, Norway). Before the tests, the roller skis were pre-warmed by 20 minutes of roller skiing on the treadmill and tested for rolling friction force (F_f_) with the towing test as previously described [4]. Skating kinematics were measured by seven Oqus infrared cameras operating at 250 Hz and Qualisys Track Manager software (Qualisys AB, Gothenburg, Sweden) using two reflective markers placed on the lateral side of the carbide tip of both poles.

Respiratory variables were measured using open-circuit indirect calorimetry (Oxycon Pro, Jaeger GmbH, Hoechberg, Germany) with calibration procedures presented previously [8]. Heart rate was continuously measured with a Polar V800 monitor (Polar Electro Oy, Kempele, Finland) and synchronized with the Oxycon Pro measurement system. Blood lactate concentration in 20 μL of blood taken from each skier’s fingertip was measured using the Biosen C-Line lactate analyser (Biosen, EKF Industrial Electronics, Magdeburg, Germany). Rating of perceived exertion (RPE) was assessed using the Borg Scale [22].

DP was performed on a modified Concept2 SkiErg (Morrisville, VT, USA) as described elsewhere [19]. Power output and cycle rate were continuously measured by the ergometer´s internal software, which has been validated in previous studies [19, 23].

Body height was determined using a calibrated stadiometer (Holtain Ltd, Crosswell, UK). Body-mass and body composition was measured using a multifrequency impedance plethysmograph body composition analyser (InBody 720, Biospace, Korea), and performed in accordance with the company´s guidelines for testing. The participants were weighed and scanned in their underwear and without shoes prior to warm-up and testing.

**Fig 1:**
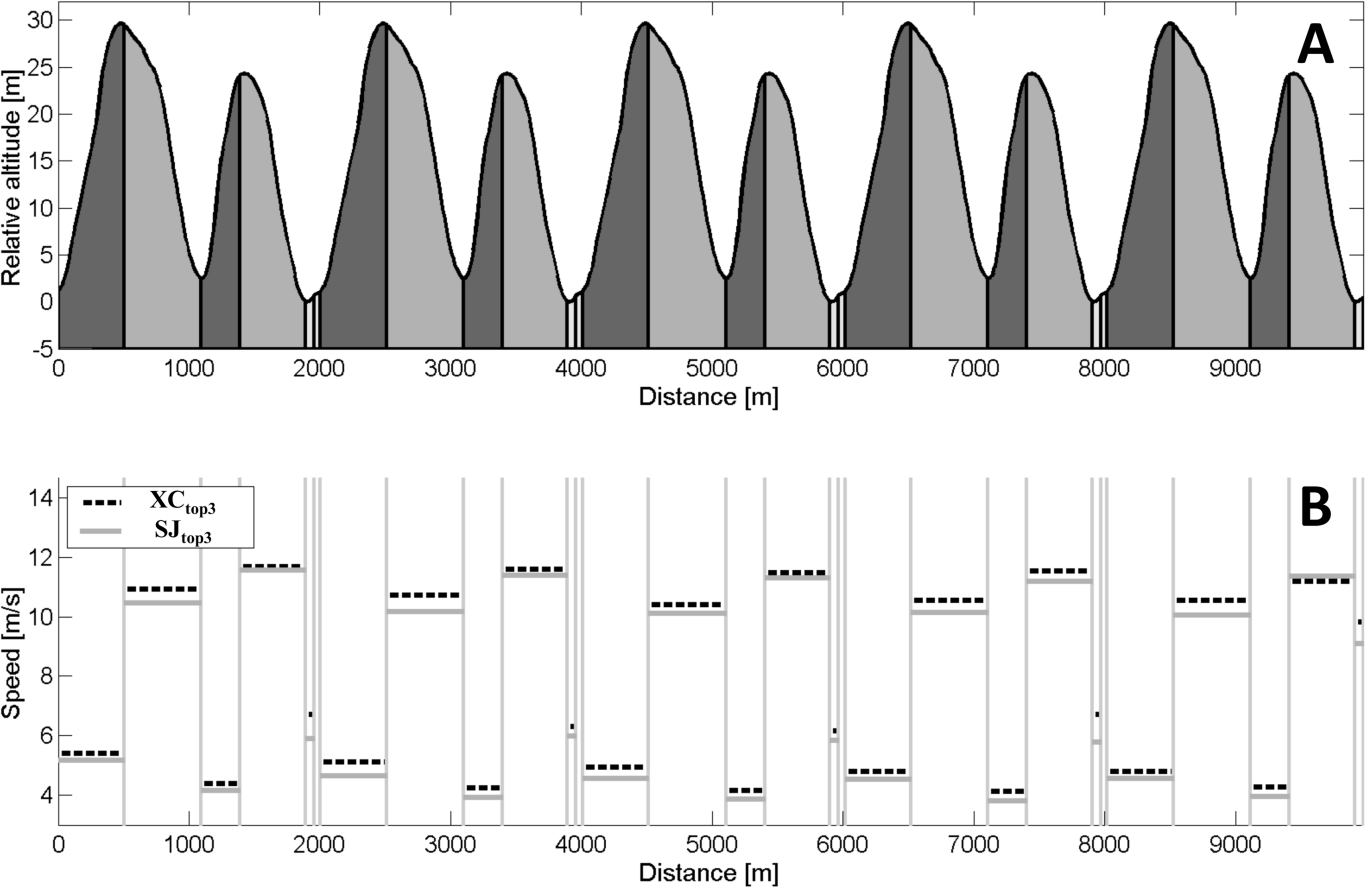
10 km race course profile with average speeds in each segment. The upper graph (A) represents the racecourse profile of the 10 km cross-country race with the relative elevation with regard to zero (start) and uphill, flat, and downhill sections in different tones of gray. The lower graph (B) represents the average speed (m·s^-1^) in the defined sections for both the top 3 ranked cross-country skiers (XC_top3_ in black stapled lines) and the top 3 ranked ski jumpers (SJ_top3_ in gray solid lines).

Valid course and elevation profiles of the XC course were standardized with a Polar V800 GPS that collected position data at a 1 Hz sampling rate with integrated barometry that collected accurate elevation data. The course was then based on the course profile divided into uphill, flat, and downhill sections that made up 40%, 5%, and 55% of the 2 km lap, respectively (Fig 1). The different sections were defined as described in a previous study [10].

During the XC race, each participant wore a Polar V800 that continuously measured their position at a 1 Hz sampling rate. All GPS watches were turned on more than 30 minutes before the race start to ensure proper GPS fixing and a low resultant inaccuracy in GPS data.

### Test protocols and measurements

#### The squat jump and ski jump imitation

Two SQJs and four IMITs were performed with the athletes´ personal jumping boots with the forefoot and rear foot placed in a standardized position on the force plates. All jumps were performed with maximal effort, and a break of 2–5 minutes between the jumps. The athletes scored each jump on a scale from 0–10, where 10 represented a perfectly executed jump. The jump with the highest rating was used for further analysis. The SQJ was performed from a stationary squat position with the hands located on the iliac crest throughout the jump, as described in a previous study [24]. The IMIT was performed from the athletes’ individually chosen ski jump position, and after maintaining this position in a static fashion for at least one second the athletes aimed to maximize their vertical lift but simultaneously gain sufficient angular momentum in order to end up in a flight-phase position to be caught by their coach, as described in a previous study [8]. The concentric push-off phase was defined as the time period of upward movement. During this phase, the vertical velocity of the centre of mass was determined by the integration of acceleration over time, which was calculated by dividing the vertical ground-reaction force with body-mass. For the IMIT, the vertical velocity was calculated at the instant of heel lift-off from the force plate (Vv_BIMIT_) as well as for maximum achieved vertical velocity (Vv_IMIT_), while only the maximum achieved vertical velocity was used for the squat jump (Vv_SQJ_). The centre of mass position was obtained through double integration of acceleration, both in horizontal and vertical directions. Ground reaction force and the position of centre of mass allowed for the calculation of angular momentum in the IMIT, which was determined both at the instant of heel lift-off (L_BIMIT_) and for the instant at maximum achieved vertical velocity (L_IMIT_).

#### The submaximal roller ski test

All athletes performed ten minutes of familiarization to the treadmill followed by one submaximal five-minute stage of treadmill skiing at 12% inclination and 7 km·h^-1^ to compare physiological response and gross efficiency. Gas exchange and heart rate were determined by the average of the last minute, and blood lactate concentration was measured directly after completion. Power output was calculated as the sum of power against gravity and friction as described previously [8]. The metabolic rate was calculated from $\dot{V}O_{2}$ and $\dot{V}CO_{2}$, as the product of $\dot{V}O_{2}$ and the oxygen energetic equivalent using the associated respiratory exchange ratio and standard conversion tables [25]. Gross efficiency was then calculated as the power output divided by the metabolic rate, and presented as a percentage.

#### The maximal roller ski test

The $\dot{V}O_{2\mathrm{peak}}$ test at 12% inclination had an initial speed of 8 km·h^-1^, which was increased by 1 km·h^-1^ every minute until exhaustion, and the highest speed maintained for at least 30 seconds was used as peak speed. $\dot{V}O_{2}$ was measured continuously, with the $\dot{V}O_{2\mathrm{peak}}$ determined by the average of the three highest 10-second consecutive measurements and according to previously determined criteria for achieving maximal effort [8]. Post-exercise blood lactate was measured one and three minutes after the test, and the highest value was used for analysis.

#### Measurements of skating kinematics

Skating kinematics were collected from the five-minute submaximal work load and the highest work load that all athletes completed during the incremental test by using Oqus infrared cameras and reflective markers on both poles. Cycle length was determined by multiplying cycle time with the belt speed of the treadmill, whereas cycle rate was calculated as the reciprocal of cycle time. Kinematical variables were collected and averaged for each athlete over 10 consecutive cycles using definitions presented previously [8].

#### Double poling all-out test

The athletes were placed in a standardized distance from a wall-mounted Concept2 ergometer, and performed the test using training shoes. The 30-s test started when the athlete performed his first pull. All athletes were instructed to double pole with full effort during the whole 30-s period.

#### Competition results

Official competition results were collected from the FIS web page (www.fis-ski.com). The hill-size of the SJ event was K-124 m where each meter jumped above or below 124 m is multiplied with 1.5 points and respectively added or subtracted from 60 pts. The total SJ pts in a competition is a summation of distance points, compensation points for wind conditions and changes in starting gate, and judges’ style points. SJ performance was defined as the sum of length points and gate/wind compensation points, thus excluding the judges´ style points to enable a better comparison with the laboratory tests. The XC performance was defined as the 10-km race time, while overall NC performance was defined as the overall competition rank in the world cup event.

The weather during the SJ event was partly cloudy with 68% humidity, air and snow temperature of respectively 2.3°C and 6.5°C, and wind conditions from 0.84 m·s^-1^ tail wind to 0.39 m·s^-1^ head wind. The average wind condition for the event was 0.23 m·s^-1^ tail wind. For the XC event, the air and snow temperature was respectively 1.8° and -2.1°Celsius with hard snow conditions.

### Statistical analysis

All data were tested for a normal distribution using a Shapiro-Wilk test as well as by visual inspection, and are presented as mean±SD (range). Accordingly, correlation analysis between laboratory and field performance was conducted using the parametric Pearson´s r or the non-parametric Spearman´s ρ correlation coefficient. Multiple regression analyses using enter-method with blocks of 1–2 independent variables were employed to predict performance in XC, SJ, and overall NC. An alpha value of 0.05 was used as the level of statistical significance. All statistical analyses were performed using SPSS 24.0 Software for Windows (SPSS Inc, Chicago, IL). To provide benchmark values of high level SJ and XC skiers among the 12 athletes participating in this study, the top 3 ranked athletes for each performance group are descriptively presented.

## Results

### Body composition and laboratory capacities

Anthropometrics and body composition for all athletes and the two performance groups are presented in Table 1, while sport-specific laboratory capacities for SJ and XC skiing are presented in Tables 2 and 3.

**Table 2:** Sport-specific capacities based on a squat jump (SQJ) and a simulated ski jump (IMIT) performed on a 3D force plate for the twelve international Nordic combined world-cup athletes and subgroups of the top 3 FIS ranked cross-country skiers (XC_top3_) and ski jumpers (SJ_top3_). All variables are presented as mean ± SD (range) for each group.

Variable	All (n = 12)	XC_top3_ (n = 3)	SJ_top3_ (n = 3)
Vv_SQJ_ (m·s^-1^)	2.73 ± 0.11 (2.60–2.92)	2.70 ± 0.05 (2.66–2.75)	2.84 ± 0.13 (2.69–2.92)
Time_IMIT_ (s)	0.41 ± 0.04 (0.30–0.46)	0.44 ± 0.03 (0.41–0.46)	0.36 ± 0.05 (0.30–0.39)
Vv_IMIT_ (m·s^-1^)	2.41 ± 0.16 (2.11–2.67)	2.25 ± 0.15 (2.11–2.40)	2.54 ± 0.13 (2.40–2.66)
VvB_IMIT_ (m·s^-1^)	1.84 ± 0.63 (0.00–2.39)	1.97 ± 0.22 (1.72–2.14)	2.15 ± 0.33 (1.77–2.39)
L_IMIT_ (N·m·s)	14.3 ± 4.1 (8.7–23.3)	15.2 ± 7.4 (8.7–23.3)	15.7 ± 2.2 (13.3–17.5)
LB_IMIT_ (N·m·s)	12.0 ± 6.5 (-3.0–22.6)	14.3 ± 7.9 (6.8–22.6)	14.6 ± 2.1 (12.3–16.5)

Vv_SQJ_ = maximum achieved vertical velocity of the skier in squat jump; Time_IMIT_ = time of push-off in the imitation jump; Vv_IMIT_ = maximum achieved vertical velocity of the skier in imitation jump; VvB_IMIT_ = vertical velocity at the instant of heel lift-off in imitation jump; L_IMIT_ = the angular momentum at the instant of maximum achieved vertical velocity in the imitation jump; LB_IMIT_ = the angular momentum at the instant of heel lift-off in the imitation jump.

**Table 3:** Physiological responses, gross efficiency, and cycle characteristics while roller ski skating at submaximal (i.e., 7 km·h^-1^) and stepwise incremental intensity to exhaustion (i.e., 12 km·h^-1^ and peak speed) on a 12% incline and performance measures of 30-seconds all-out double poling in the twelve international Nordic combined world-cup athletes and subgroups of the top 3 FIS ranked cross-country skiers (XC_top3_) and ski jumpers (SJ_top3_). All variables are presented as mean ± SD (range) for each group.

Variable	All (n = 12)	XC_top3_ (n = 3)	SJ_top3_ (n = 3)
7 km·h^-1^			
$\dot{V}O_{2}$ (ml·kg^-1^·min^-1^)	50.0 ± 1.8 (47.5–53.7)	48.9 ± 1.0 (47.8–49.7)	49.0 ± 1.6 (57.5–50.6)
$\dot{V}O_{2}$ (L·min^-1^)	3.29 ± 0.31 (2.68–3.73)	3.38 ± 0.20 (3.16–3.54)	2.91 ± 0.27 (2.68–3.21)
$\dot{V}O_{2}$ in % of $\dot{V}O_{2\mathrm{peak}}$	68.2 ± 4.2 (61.5–75.4)	63.9 ± 2.4 (62–66)	67.2 ± 3.1 (65–71)
HR in % of HR_peak_	80.9 ± 5.2 (72–90)	77.0 ± 5.0 (72–81)	80.8 ± 4.5 (76–85)
RER	0.90 ± 0.05 (0.82–0.98)	0.89 ± 0.03 (0.86–0.92)	0.93 ± 0.04 (0.89–0.97)
BLa (mmol·L^-1^)	2.5 ± 0.6 (1.6–3.4)	2.1 ± 0.1 (2.0–2.2)	2.6 ± 0.3 (2.2–2.8)
GE (%)	16.2 ± 0.5 (15.4–16.9)	16.6 ± 0.3 (16.3–16.8)	16.4 ± 0.5 (16.0–16.9)
RPE (6–20)	11.9 ± 1.38 (10–14)	13.0 ± 0.0 (13)	11.3 ± 1.5 (10–13)
Cycle length (m)	2.79 ± 0.18 (2.51–3.04)	2.88 ± 0.14 (2.76–3.04)	2.72 ± 0.18 (2.55–2.91)
Cycle rate (Hz)	0.70 ± 0.05 (0.64–0.77)	0.68 ± 0.04 (0.64–0.71)	0.72 ± 0.05 (0.67–0.76)
12^a^ km·h^-1^			
Cycle length (m)	3.96 ± 0.37 (3.0–4.3)	3.99 ± 0.12 (3.87–4.11)	3.92 ± 0.35 (3.60–4.29)
Cycle rate (Hz)	0.85 ± 0.09 (0.77–1.10)	0.84 ± 0.03 (0.81–0.86)	0.85 ± 0.07 (0.78–0.93)
Peak speed			
Peak speed (km·h^-1^)	12.9 ± 0.5 (12–14)	13.3 ± 0.6 (13–14)	13.0 ± 0.0 (13)
$\dot{V}O_{2\mathrm{peak}}$ (ml·kg^-1^·min^-1^)	73.5 ± 4.3 (66.9–80.8)	76.6 ± 4.4 (72.1–80.8)	73.0 ± 1.5 (71.5–74.5)
$\dot{V}O_{2\mathrm{peak}}$ (L·min^-1^)	4.83 ± 0.50 (4.12–5.75)	5.30 ± 0.42 (4.94–5.75)	4.33 ± 0.21 (4.12–4.54)
Peak RER	1.17 ± 0.06 (1.03–1.25)	1.18 ± 0.00 (1.18)	1.18 ± 0.05 (1.15–1.23)
Peak VE (L·min^-1^)	157.0 ± 11.6 (133–172)	157.3 ± 5.1 (153–163)	155.3 ± 13.6 (140–166)
Peak bLa (mmol·L^-1^)	13.1 ± 1.6 (10.2–15.2)	13.9 ± 0.8 (13.4–14.8)	12.9 ± 0.7 (12.3–13.7)
30-s all out DP exercise			
Mean power output			
(W)	323 ± 46 (233–379)	344 ± 39 (316–371)	285 ± 55 (233–343)
(W·kg^-1^)	4.9 ± 0.4 (4.1–5.6)	5.1 ± 0.2 (4.9–5.2)	4.8 ± 0.6 (4.1–5.4)
(W·LM^-1^)	5.3 ± 0.4 (4.5–6.0)	5.4 ± 0.2 (5.2–5.5)	5.1 ± 0.6 (4.5–5.7)
(W·UB LM^-1^)	9.6 ± 0.7 (8.4–10.7)	9.9 ± 0.2 (9.7–10.0)	9.3 ± 1.0 (8.4–10.3)
Mean cycle rate (Hz)	1.38 ± 0.15 (1.18–1.63)	1.33 ± 0.07 (1.28–1.38)	1.59 ± 0.06 (1.52–1.63)

$\dot{V}O_{2}$ = oxygen uptake; HR = heart rate; RER = respiratory exchange ratio; BLa = blood lactate concentration; GE = gross efficiency; RPE = rating of perceived exertion; $\dot{V}O_{2\mathrm{peak}}$ = peak oxygen uptake from incremental test to exhaustion; VE = ventilation.

^a^ 12 km·h^-1^ was the highest speed completed by all 12 athletes in the incremental test.

### Competition results

Competition results for all athletes and the two performance groups are presented in Table 4. All 6 athletes in SJ_top3_ and XC_top3_ finished in top 7 of the athletes recruited to this study. Although they differed substantially in their XC and SJ performance, the mean overall ranking and time difference to the winner of the NC competition was close to identical.

**Table 4:** Ski jumping (SJ), cross-country (XC) skiing, and overall result of the world-cup event, with the percentage of the total XC race time spent in uphill, flat, and downhill sections, in twelve international Nordic combined world-cup athletes (n = 12) and subgroups of the top 3 FIS ranked athletes in cross-country skiing (XC_top3_) and ski jumping (SJ_top3_). All variables are presented as mean ± SD (range) for each group.

Variable	All (n = 12)	XC_top3_ (n = 3)	SJ_top3_ (n = 3)
SJ result			
Points	57.2 ± 8.3 (46.7–70.0)	49.2 ± 1.37 (48.0–50.7)	67.4 ± 2.4 (65.3–70.0)
Rank	29.5 ± 13.9 (5–45)	41.7 ± 2.5 (39–44)	11.3 ± 6.5 (5–18)
XC result			
Minutes	25.00 ± 1.07 (23.55–27.08)	23.81 ± 0.30 (23.55–24.13)	25.24 ± 0.61 (24.57–25.77)
% uphill	60.8 ± 0.9 (59.5–62.2)	60.1 ± 0.6 (59.5–60.6)	60.8 ± 0.7 (59.9–61.3)
% flat	4.1 ± 0.2 (3.9–4.4)	4.1 ± 0.1 (3.9–4.2)	4.3 ± 0.2 (4.1–4.4)
% downhill	33.8 ± 0.8 (32.6–35.2)	34.6 ± 0.6 (34.0–35.2)	33.5 ± 0.3 (33.3–33.8)
Rank	23.0 ± 16.7 (1–45)	3.3 ± 3.2 (1–7)	28.0 ± 13.5 (13–39)
Overall result			
Minutes	26.39 ± 0.94 (24.96–28.54)	25.82 ± 0.20 (25.62–26.01)	25.74 ± 0.68 (24.96–26.20)
Rank	28.0 ± 11.5 (7–45)	19.7 ± 5.5 (14–25)	20.0 ± 11.3 (7–27)

### Correlation and regression analysis

**Fig 2:**
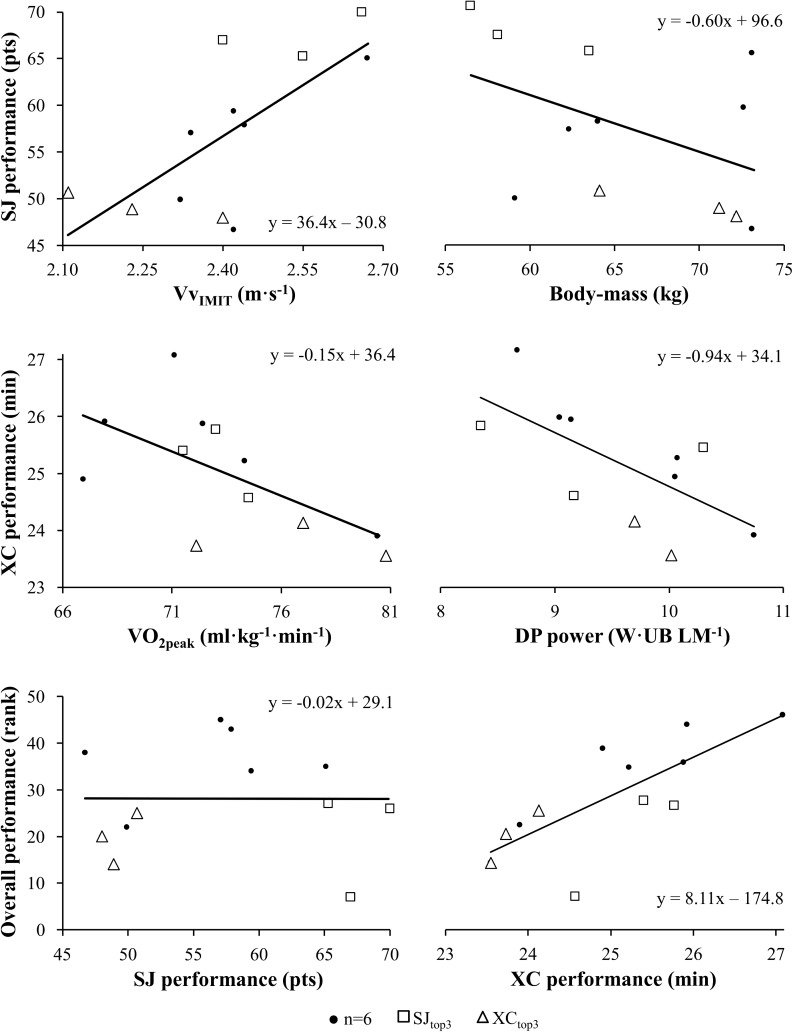
Ski jumping (SJ) and cross-country (XC) performance in relationship to the sport-specific capacities that combined best explained the variance in performance, as well as overall performance in relationship to the SJ and XC performance for the 12 international Nordic combined world-cup athletes. The data points represent the top 3 ranked ski jumpers (□ SJ_top3_), top 3 ranked XC skiers (Δ XC_top3_), and the remaining 6 athletes of the study (● n = 6). The lines were obtained by linear regression. Vv_IMIT_ = maximum achieved vertical velocity of the skier in imitation jump; $\dot{V}O_{2\mathrm{peak}}$ = peak oxygen uptake from incremental test to exhaustion; DP = double poling; UB LM = upper-body lean-mass.

Correlations between laboratory variables and XC, SJ, and overall performance are listed in Table 5, while the most central associations are presented in Fig 2. For the specific sections, time spent uphill correlated significant with body-mass-normalized $\dot{V}O_{2\mathrm{peak}}$ (r = -0.633, p = 0.027).

**Table 5:** Pearson’s r or Spearman’s ρ correlations between field performance and laboratory capacities in twelve international Nordic combined world-cup athletes.

	XC performance (time)	SJ performance (pts)	Overall performance (rank)
**Field performance (n = 12)**			
Time uphill	**.980**^#^ **(p<0.001)**		
Time downhill	**.847**^#^ **(p<0.001)**		
Time flat	**.774**^#^ **(p<0.001)**		
XC performance		.565 (p = 0.055)	.**757**^#^ **(p = 0.004)**
SJ performance	.565 (p = 0.055)		-.013 (p = 0.967)
SJ in-run speed (km·h^-1^)		.200 (p = 0.533)	.064 (p = 0.844)
**SJ specific variables (n = 12)**			
Body mass (kg)	-.119^a^ (p = 0.712)	-.511^a^ (p = 0.089)	.270^a^ (p = 0.397)
Body mass index (kg·m^-2^)	-.481 (p = 0.113)	-.426 (p = 0.168)	-.052 (p = 0.872)
Vv_SQJ_ (m·s^-1^)	.237 (p = 0.458)	.528 (p = 0.078)	.042 (p = 0.897)
Time_IMIT_ (s)	**-.605**^a^* **(p = 0.037)**	**-.763**^a^^#^ **(p = 0.004)**	-.186 (p = 0.562)
Vv_IMIT_ (m·s^-1^)	.525 (p = 0.080)	.711* **(p = 0.010)**	.238 (p = 0.456)
VvB_IMIT_ (m·s^-1^)	.224^a^ (p = 0.484)	.329^a^ (p = 0.297)	-.035^a^ (p = 0.914)
**XC specific variables (n = 12)**			
$\dot{V}O_{2\mathrm{peak}}$ (L·min^-1^)	-.511 (p = 0.090)	-.519 (p = 0.084)	-.164 (p = 0.611)
$\dot{V}O_{2\mathrm{peak}}$ (ml·kg^-1^·min^-1^)	-.619* (p = 0.032)	-.192 (p = 0.550)	-.654* (p = 0.021)
DP power (W)	-.389 (p = 0.237)	-.563 (p = 0.072)	.074 (p = 0.829)
DP power (W·kg^-1^)	-.608* (p = 0.047)	-.548 (p = 0.081)	-.243 (p = 0.472)
DP power (W·LM^-1^)	-.607* (p = 0.048)	-.617* (p = 0.043)	-.178 (p = 0.602)
DP power (W·UB LM^-1^)	-.671* (p = 0.024)	-.568 (p = 0.069)	-.253 (p = 0.452)
GE 7 km/h (%)	-.315 (p = 0.319)	-.081 (p = 0.802)	-.305 (p = 0.335)
CL 7 km/h (m)	-.194 (p = 0.545)	-.427 (p = 0.167)	-.008 (p = 0.980)
CL 12 km/h (m)	-.084^a^ (p = 0.795)	-.266^a^ (p = 0.404)	.350^a^ (p = 0.265)

XC = cross-country; SJ = ski jumping; Vv_SQJ_ = maximum achieved vertical velocity of the skier in squat jump; Time_IMIT_ = time of push-off in the imitation jump; Vv_IMIT_ = maximum achieved vertical velocity of the skier in imitation jump; VvB_IMIT_ = vertical velocity at the point of heel lift-off in imitation jump; $\dot{V}O_{2\mathrm{peak}}$ = peak oxygen uptake from incremental test to exhaustion; DP = double poling; UB = upper-body; LM = lean-mass; GE = gross efficiency; CL = cycle length.

^a^ Spearman’s ρ correlation

*p<0.05

^#^p<0.01

The regression analyses, with the various laboratory capacities and anthropometric characteristics as independent variables, resulted in the following three equations as the best predictions for SJ (I), XC (II), and overall (III) performance respectively.

$$\begin{array}{l} \mathrm{SJ performance}=8.51+35.90\cdot {\mathrm{Vv}}_{\mathrm{IMIT}}\;(m\cdot s^{-1})-0.58\cdot \mathrm{body-mass}\;(\mathrm{kg})\\ \;(F_{2,9}=10.41,\;p<0.01) \end{array}$$ (I)

The factors included in Eq (I) all significantly contributed to model I (all p<0.05) which explained 70% of the variance in SJ performance.

$$\begin{array}{l} \mathrm{XC performance}=40.29-0.12\cdot \dot{V}O_{2\mathrm{peak}}\;(\mathrm{ml}\cdot {\mathrm{kg}}^{-1}\cdot \min^{-1})-0.64\cdot \mathrm{DP power}\;(W\cdot {\mathrm{UB LM}}^{-1})\\ \;(F_{2,8}=8.63,p=0.01) \end{array}$$ (II)

$\dot{V}O_{2\mathrm{peak}}$ significantly contributed to model II (p<0.05), while DP power showed a tendency (p = 0.07). Model II explained 68% of the variance in XC performance.

$$\begin{array}{l} \mathrm{Overall performance}=156.45-1.75\cdot \dot{V}O_{2\mathrm{peak}}\;(\mathrm{ml}\cdot {\mathrm{kg}}^{-1}\cdot \min^{-1})\\ \;(F_{1,10}=7.47,\;p=0.02) \end{array}$$ (III)

Model III explained 43% of the variance in overall performance.

## Discussion

The present study investigated associations between sport-specific laboratory capacities and NC world-cup performance in NC athletes who combine well-developed explosiveness and SJ technique with aerobic energy delivery capacity and XC skiing efficiency. Our main findings were as following: 1) vertical velocity obtained in an imitation jump (Vv_IMIT_) and body-mass provided the best prediction of SJ performance; 2) body-mass-normalized $\dot{V}O_{2\mathrm{peak}}$ and double poling (DP) power provided the best prediction of XC performance; 3) body-mass-normalized $\dot{V}O_{2\mathrm{peak}}$ was the only significant correlate with overall NC performance. In addition, the benchmark values provided for the best performing athletes in SJ and XC skiing among NC athletes further support the importance of these factors for the specific events.

SJ performance correlated significantly with both Vv_IMIT_ and time_IMIT_, while Vv_IMIT_ together with body-mass were best suited to predict SJ performance. These findings are in accordance with established performance characteristics of successful ski jumpers, where the ability to reach maximal vertical velocity in a very short time (<0.35 s) is necessary for a successful take-off [2, 5]. This is, however, the first study to show that the same variables correlate to SJ performance in NC, where NC athletes possess some different challenges than specialist ski jumpers. The time available at the take-off in the SJ hill may present a greater challenge for NC athletes than specialist ski jumpers as two-thirds of the NC athletes’ annual training consists of endurance training [8, 24]. This does not only leave less time available for power and SJ specific training compared to the specialists, but endurance training may lead to negative effects on muscle strength and power [26, 27]. This might partly explain the correlation found between Vv_IMIT_ or time_IMIT_ with XC performance. Furthermore, the lack of association between Vv_SQJ_ and SJ performance suggests that the maximum vertical velocity achieved in the technically challenging task of an IMIT is more relevant for SJ performance than the pure vertical jump capacity assessed by SQJ.

Although body-mass coupled with Vv_IMIT_ gave the best prediction of SJ performance, neither body-mass nor BMI alone showed a significant correlation with SJ performance. This lack of association, however, might be influenced by the two-sided effect of body-mass. While a lower body-mass will reduce the effect of gravity during the flight phase, and hence have a positive impact on performance, it will also reduce the positive effect of gravity on in-run speed and the horizontal momentum at take-off [5]. Yet, a low body-mass has been found to have an overall positive effect on SJ performance in simulation studies [5, 28], in addition to being beneficial for maximizing vertical velocity at take-off. Hence, the overall assessment is that low body-mass is a contributing factor for SJ performance. This is also in agreement with a low body-mass being a performance characteristic found among successful ski jumpers [8, 9].

As expected, body-mass-normalized $\dot{V}O_{2\mathrm{peak}}$ and DP power were the best predictors for XC performance. The importance of a high aerobic capacity is well established in several endurance sports, including XC skiing [3, 16], but this is the first study to validate the association to competitive performance among elite NC athletes. The importance of upper-body power for XC performance is repeatedly shown in recent XC skiing literature [17, 20, 23, 29, 30]. The finding that DP power significantly correlated with XC performance in this study was therefore no surprise. In our case, the highest correlation was when normalizing power for upper-body lean-mass. The latter is of particular interest for the NC athlete, as the upper-body power capacity must be balanced with a low body-mass to optimize SJ performance.

In contrast to established performance characteristics among elite XC skiers [3, 4, 17], no correlation between neither submaximal gross efficiency nor cycle length with XC performance was found here. The large variation in body-mass-normalized $\dot{V}O_{2\mathrm{peak}}$ found in the current study, ranging from 66.9 to 80.8 ml·kg^-1^·min^-1^, may result in gross efficiency being a less important performance measure for XC skiing among elite NC athletes compared to XC skiers with more homogenous $\dot{V}O_{2\mathrm{peak}}$ levels. Also the lack of association between cycle length and XC performance may be related to the heterogeneous study group; for example in cycling, variation in muscle fiber type distribution has been found impact the energetically optimal cadence [31]. However, as we do not have muscle biopsy of these athletes, this is something future studies need to investigate.

Since the athletes’ capacities were tested in a laboratory setting, some of the constraints are clearly different than the performance settings measured outdoors on snow. For example, the jump capacity for SJ is measured using full friction forces during push-off while the actual ski jump is executed while gliding in high speed on ice tracks with close to zero friction. Hence, the laboratory test enables the skier to employ a movement strategy that is not fully possible to perform at the take-off in the jumping hill [32]. In the XC roller ski test, the roller skis are shorter than skis and the wheels have different rolling friction and push-off mechanics. This may allow for slightly different technical strategies compared to on-snow skiing, which may especially have an impact on the gross efficiency measure. However, the scope of this study was indeed to elucidate the association of laboratory capacities in sport specific movement techniques used for monitoring athletes’ development during the training year and field performance. Hence, in-depth technique comparisons of laboratory versus field SJ and XC skiing should be investigated in follow-up studies.

Of the sport-specific laboratory determinants investigated in this study, body-mass-normalized $\dot{V}O_{2\mathrm{peak}}$ alone best predicted overall NC performance. This can largely be explained by the fact that in this specific event, XC performance had a significant correlation with overall NC performance while SJ performance did not. In addition, a high body-mass-normalized $\dot{V}O_{2\mathrm{peak}}$ is influenced both by the absolute $\dot{V}O_{2\mathrm{peak}}$ and body-mass, which separately were shown as important determinants for XC skiing and SJ performance, respectively. From a general perspective, it is rather unique that NC athletes with explosiveness close to the upper human limits are able to obtain $\dot{V}O_{2\mathrm{peak}}$ values as high as 80 ml·kg^-1^·min^-1^. Whether the impact of SJ versus XC performance, and the associations to laboratory capacities, on the overall NC result apply to other venues and conditions (i.e. wind, snow, etc.) need to be investigated further. Although a definite conclusion cannot be made from this study, it constitutes an important point-of departure for future studies on the sport of NC.

## Conclusion

Vertical IMIT velocity and body-mass in combination best predicted SJ performance, whereas body-mass normalized $\dot{V}O_{2\mathrm{peak}}$ and upper-body power best predicted XC skiing performance. The test capacities provided for the best SJ and XC skiers among our 12 NC athletes may serve as reference values for world-class performance in these events. Specifically, the 3 best SJ obtained a group mean of ~2.5 m·s^-1^ vertical velocity in the imitation jump with a body-mass of <60 kg, with the respective values being 12–15% different among the 3 best XC skiers. Interestingly, there was only 5% difference in vertical velocity in the squat jump between the two performance groups, which indicates that performance in the sport-specific movement of an imitation jump distinguishes performance groups more than pure vertical jump capacity. The 3 best XC skiers showed a group mean of >76 ml·kg^-1^·min^-1^ in $\dot{V}O_{2\mathrm{peak}}$ and upper-body power of 344 W and 5.1 W·kg^-1^, being respectively 5%, 21%, and 6% higher than the 3 best SJ.

Overall, the concurrent development of $\dot{V}O_{2\mathrm{peak}}$, upper-body power, and SJ-specific vertical jump capacity while minimizing body-mass within the BMI limit set by FIS should be considered in the seasonal training of NC athletes.
